# LASP1 is a novel BCR-ABL substrate and a phosphorylation-dependent binding partner of CRKL in chronic myeloid leukemia

**DOI:** 10.18632/oncotarget.2072

**Published:** 2014-06-07

**Authors:** Jochen J. Frietsch, Carolin Kastner, Thomas G. P. Grunewald, Hardy Schweigel, Peter Nollau, Janine Ziermann, Joachim H. Clement, Paul La Rosée, Andreas Hochhaus, Elke Butt

**Affiliations:** ^1^ Klinik für Innere Medizin II, Abteilung Hämatologie und internistische Onkologie, Universitätsklinikum Jena, Jena, Germany; ^2^ Institute for Clinical Biochemistry and Pathobiochemistry, University Clinic of Wuerzburg, Wuerzburg, Germany; ^3^ INSERM Unit 830, Genetics and Biology of Cancers, Institute Curie Research Center, Paris, France; ^4^ Department of Clinical Chemistry, University Medical Center Hamburg-Eppendorf, Hamburg, Germany; ^5^ Research Institute Children's Cancer Center and Clinic of Pediatric Hematology and Oncology, University Medical Center Hamburg-Eppendorf, Hamburg, Germany

**Keywords:** LASP1, CML, BCR-ABL, CRKL, nilotinib

## Abstract

Chronic myeloid leukemia (CML) is characterized by a genomic translocation generating a permanently active BCR-ABL oncogene with a complex pattern of atypically tyrosine-phosphorylated proteins that drive the malignant phenotype of CML. Recently, the LIM and SH3 domain protein 1 (LASP1) was identified as a component of a six gene signature that is strongly predictive for disease progression and relapse in CML patients. However, the underlying mechanisms why LASP1 expression correlates with dismal outcome remained unresolved.

Here, we identified LASP1 as a novel and overexpressed direct substrate of BCR-ABL in CML. We demonstrate that LASP1 is specifically phosphorylated by BCR-ABL at tyrosine-171 in CML patients, which is abolished by tyrosine kinase inhibitor therapy. Further studies revealed that LASP1 phosphorylation results in an association with CRKL – another specific BCR-ABL substrate and *bona fide* biomarker for BCR-ABL activity. pLASP1-Y171 binds to non-phosphorylated CRKL at its SH2 domain. Accordingly, the BCR-ABL-mediated pathophysiological hyper-phosphorylation of LASP1 in CML disrupts normal regulation of CRKL and LASP1, which likely has implications on downstream BCR-ABL signaling. Collectively, our results suggest that LASP1 phosphorylation might serve as an additional candidate biomarker for assessment of BCR-ABL activity and provide a first step toward a molecular understanding of LASP1 function in CML.

## INTRODUCTION

Chronic myeloid leukemia (CML) is a hematopoietic stem cell disease, characterized by clonal expansion of differentiated cells. CML is driven by the Philadelphia (Ph) chromosome, which results in the generation of the BCR-ABL fusion oncogene and expression of the constitutively active BCR-ABL tyrosine kinase. As a consequence, the deregulated kinase phosphorylates substrates such as ERK and STAT5 and causes activation of RAS/MAPK/ERK downstream signaling and the PI3K/AKT pathway, which ultimately promotes cell proliferation and suppresses apoptosis [1, 2].

Abnormal integrin function is based on BCR-ABL phosphorylation of cytoskeleton proteins such as paxillin, talin, focal adhesion kinase 2 (FAK2), and CRKL (Crk-like) [3, 4]. CRKL is an adaptor protein and the major substrate of BCR-ABL in CML cells [5]. The phosphorylation status of CRKL has been used as a marker to predict the efficacy of tyrosine kinase inhibitors (TKI; e.g. imatinib and nilotinib) in the treatment of CML patients [6, 7]. In this context, we wondered whether other established physiological ABL kinase substrates are pre-phosphorylated in leukemia cells. A known substrate of ABL is the LIM and SH3 domain protein LASP1 [8]. Interestingly, Yeung et al. recently identified LASP1 as one out of six signature genes that are highly predictive for CML disease phases, which enable a more accurate prediction of relapse after stem cell transplantation than clinical risk factors alone [9].

LASP1 was initially identified from a cDNA library of breast cancer metastases and predominantly localizes at sites of actin assembly, such as focal contacts, lamellipodia and membrane ruffles [10, 11], where it interacts with a number of motility associated proteins such as zyxin, dynamin, lipoma preferred partner (LPP), actin, paladin and kelch related protein 1 (KRP1) [10]. Phosphorylation at Tyr-171 by ABL is seen in apoptotic cells and specifically blocks LASP1 translocation to sites of focal adhesions, i.e. cell-matrix and cell-cell contacts [8]. Although LASP1 is expressed at low basal levels in virtually all normal human tissues [10], protein overexpression has been observed in several cancer entities e.g. breast, ovarian, colon, prostate, liver, and bladder carcinoma as well as medulloblastoma [12-17]. Furthermore, LASP1 expression and nuclear localization significantly correlates with poor outcome of cancer patients [11, 12, 15, 18]. Silencing of LASP1 by RNA interference in various cell lines results in a strong inhibition of proliferation and migration with cell-cycle arrest in G2/M phase, whereas overexpression increased cancer cell proliferation and cell motility [13, 19].

As the oncogenic BCR-ABL tyrosine kinase is constitutively active, LASP1 may not only be phosphorylated in apoptotic cells, but also in defined Ph chromosome positive CML cells. Here, we integrated comprehensive transcriptomic and biochemical data demonstrating that LASP1 is highly overexpressed and specifically phosphorylated at tyrosine-171 by BCR-ABL in CML patients. Moreover, we identified LASP1 as a novel binding partner of CRKL and elucidated the mode of this protein-protein-interaction. Collectively, our results suggest that LASP1 phosphorylation might serve as a candidate biomarker for measurement of BCR-ABL activity and point to an important role of LASP1 in aberrant BCR-ABL signaling. Moreover, these data provide a first mechanistic insight in why LASP1 overexpression might contribute to CML progression.

## RESULTS

### LASP1 is overexpressed in CML

**Figure 1:**
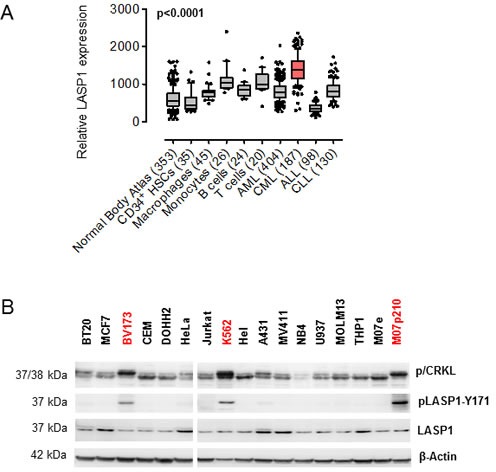
LASP1 is overexpressed in CML and phosphorylated at Tyr-171 in leukemia cell lines (A) Microarray analysis of LASP1 (3927_at) in CML compared to normal tissues, normal bone marrow-derived cells, and myeloid and lymphoblastic leukemias (AML, acute myeloid leukemia; cALL, common acute lymphoblastic leukemia; CLL, *chronic lymphocytic leukemia), Mann-Whitney test.* (B) Western blot analysis (gradient gel) of phospho/dephospho CRKL (p/CRKL), LASP1 and pLASP1-Y171 in different cancer cell lines (breast cancer: BT-20, MCF-7; cervical cancer: HeLa; epidermoid cancer: A-431; monocytes-macrophages: U-937; acute myeloid leukemia: MOLM-13; acute promyelocytic leukemia: NB4; acute monocytic leukemia MV4-11, THP-1; erythroleukemia: HEL; megakaryoblastic leukemia: M07e; T-cell leukemia: Jurkat; B-cell lymphoma: DOHH-2; acute lymphoblastic leukemia: CCRF-CEM; chronic myeloid leukemia: K562, BV173). Cell lines harboring BCR-ABL are marked red. β-actin served as a loading control.

Known to be overexpressed in several cancer entities [12-16, 19], we sought to investigate the LASP1 gene expression pattern in publicly available microarray datasets for leukemia and normal tissues. Specifically, microarray data of an established normal body atlas (n = 353) [20] normal CD34^+^ HSCs (n = 35) [21], macrophages (n = 45), monocytes (n = 26), B cells (n = 24), and T cells (n = 20) were compared with those for four major lymphoma and leukemia entities (n = 819). As displayed in Figure 1A, LASP1 is significantly (p < 0.0001) and highly overexpressed in CML cells compared to all normal tissues and cells, respectively, while it is considerably lower expressed in cells derived from acute myeloid leukemia (AML), common acute lymphoblastic leukemia (cALL) or *chronic lymphocytic leukemia* (CLL).

To test a possible link between LASP1 and the BCR-ABL tyrosine kinase that is the predominant driver mutation in CML cells, we subjected BCR-ABL positive (K562, BV173, M07p210) and BCR-ABL negative cell lines for LASP1 expression and pLASP1-Y171 phosphorylation, the known phosphorylation site for ABL kinase [8]. As shown in Figure 1B, LASP1 is expressed in all cancer cell lines tested. However, only the leukemia cell lines harboring Ph chromosome with constitutively active BCR-ABL (marked red) show a strong phosphorylation of LASP1 at tyrosine-171. This effect is most obvious when comparing the BCR-ABL negative M07e cell line that shows no pLASP1-Y171 with the derivative BCR-ABL positive cell line M07p210 that shows marked LASP1-Y171 phosphorylation.

### LASP1 is phosphorylated at tyrosine 171 in CML patients

**Figure 2:**
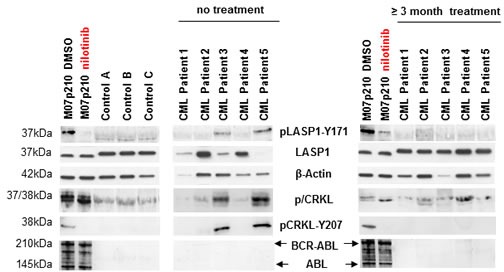
LASP1 is phosphorylated in CML patients Western blot analysis (gradient gel) of LASP1, pLASP1-Y171 and p/CRKL in the leukemia cell line M07p210, in three healthy control donors and in five CML patients before and after TKI treatment. Loading M07p210: 10 μg; loading blood samples: 10 μg for LASP1 and β-actin, 100 μg for pLASP1-Y171, CRKL and BCR-ABL WB. Expression of ABL is not reliably detectable in CML patients. LASP1 is phosphorylated only in CML patients and in the M07p210 cell line but not in controls. In general, CRKL and pLASP1-Y171 phosphorylation decrease under TKI therapy. Patient 4 shows only minimal cytogenetic response to nilotinib treatment (Table 1).

Using anti-phosphotyrosine immunoblotting, we compared the phosphorylation pattern of LASP1 from 3 healthy donors with 5 CML patients before and 12 weeks after commencing treatment with the tyrosine kinase inhibitors (TKIs) nilotinib and ponatinib [22, 23]. As a control, we probed for CRKL, the most prominent BCR-ABL substrate [7]. The non-phosphorylated CRKL (37 kDa) migrates faster in SDS-PAGE than its phosphorylated form (38 kDa). This is most obvious in the BCR-ABL positive cell line M07p210. As expected, CRKL is phosphorylated in CML patients (Figure 2, pCRKL band at 38 kDa) while in control blood samples from healthy donors, pCRKL was not detected. Likewise, we tested for LASP1 phosphorylation by BCR-ABL kinase with a specific antibody against pLASP1-Y171 (Supplementary Figure 1), the known ABL kinase phosphorylation site in LASP1. Although present in similar protein concentrations, LASP1 is phosphorylated at that site only in CML patients and in the M07p210 cell line but not in controls. After 12 weeks of therapy with nilotinib or ponatinib, both, CRKL phosporylation and LASP1-Y171 phosphorylation are impaired and no longer detectable by Western blot in CML patients that responded to TKI therapy (Figure 2) (for detailed patient characteristics, see Table 1). A similar effect is observed in M07p210 cells after 24 h treatment with 160 nM nilotinib (Figure 2, very left).

**Table 1:** Patients characteristics

	Patient 1	Patient 2	Patient 3	Patient 4	Patient 5
Sex	Male	Male	Male	Female	Male
Age at diagnosis, years	23	57	59	54	63
EUTOS score	Low risk (42)	Low risk (49)	Low risk (54)	Low risk (14)	Low risk (14)
Initial ratio BCR-ABL/ABL (%)	50	45	52	62	50
Initial WBC (/nl)	112.3	28.4	394.8	55.9	43.1
BCR-ABL transcript type	b2a2	b3a2	b2a2	b3a2	b3a2
Therapy	nilotinib	nilotinib	nilotinib	nilotinib	ponatinib
3 month BCR-ABL (IS)	0.008	0.013	0.055	20.5	0.263
3 month WBC (/nl)	4.2	5.1	6.7 *	13.1 *	13.1
Response at 3 months	MMR	MMR	MMR *	MinCyR *	n.d.

MMR, major molecular response; MinCyR, minimal cytogenetic response; IS, international scale; WBC, white blood cells;

* 6 month; n.d., not detected after 3 months.

Patient 4 achieved only minimal cytogenetic response (minCyR) under nilotinib treatment. While LASP1 phoshorylation was absent, CRKL phosphorylation, however, was still detectable (Figure 2) suggesting different BCR-ABL substrate specificities for both proteins.

Together, these data suggest that LASP1 is, like CRKL, a marker for BCR-ABL kinase activity in CML.

### LASP1 is a substrate of BCR-ABL

**Figure 3:**
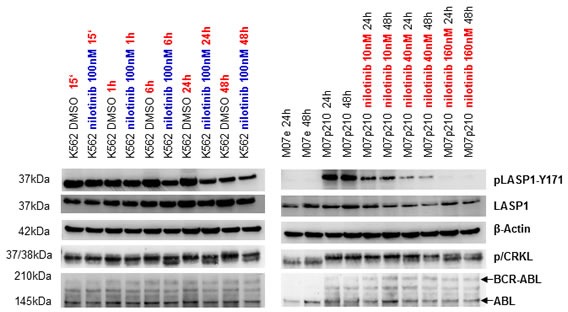
Phosphorylation of LASP1 and CRKL is inhibited by the tyrosine kinase inhibitor nilotinib Western blot analysis (gradient gel) of BCR-ABL, ABL, LASP1, pLASP1-Y171 and p/CRKL in the leukemia cell lines K562 and M07p210 after a time- and concentration-dependent treatment with the tyrosine kinase inhibitor nilotinib. CRKL: (37 kDa); pCRKL: phospho-CRKL (38 kDa). In the non-transformed cell line M07e, no BCR-ABL kinase is present; thus no CRKL and LASP1 phosphorylation is observed. β-actin served as loading control.

To further investigate pLASP1-Y171 phosphorylation, we used the cell line K562 (derived from a patient during blast crisis), as well as wild-type M07e and BCR-ABL transformed M07p210 cell lines. Cells were treated with either 100 nM nilotinib at various time points or with 10 nM, 40 nM and 160 nM of nilotinib for 24h and 48h. DMSO treated cells served as control. As seen in Figure 3, LASP1 and CRKL are only phosphorylated in the BCR-ABL expressing cell lines K562 (left panel) and M07p210 but not in the BCR-ABL negative M07e cells (right panel) Both proteins are dephosphorylated in a time- and concentration-dependent manner after nilotinib treatment (Figure 3). Similar results were obtained with the human CML cell line BV173 (data not shown).

**Figure 4:**
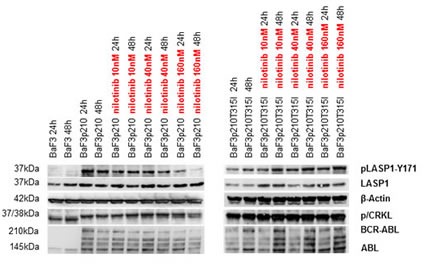
LASP1 and CRKL are phosphorylated by BCR-ABL-kinase Western blot analysis (gradient gel) of BCR-ABL, ABL, LASP1, pLASP1-Y171 and p/CRKL in Ba/F3 leukemia cell lines. While CRKL and LASP1 phosphorylation is inhibited by nilotinib in the BCR-ABL expressing cell line Ba/F3p210, inhibition failed in the nilotinib resistant BCR-ABL mutant cell line Ba/F3p210T315I. CRKL: (37 kDa); pCRKL: phospho-CRKL (38 kDa). In the non-transformed cell line Ba/F3, no BCR-ABL kinase is present; thus no CRKL and LASP1 phosphorylation is observed. β-actin is shown for loading control.

To further exclude LASP1 phosphorylation by physiologically expressed ABL kinase (see ABL kinase Western blots in Figures 3 and 4), we validated these experiments with the murine lymphoid cell line Ba/F3, the BCR-ABL positive variant of Ba/F3p210 and its TKI-resistant mutant Ba/F3p210^T315I^. As anticipated from the results with M07e and M07p210 (Figure 3), only the BCR-ABL expressing cell lines Ba/F3p210 and Ba/F210^T315I^ revealed phosphorylation of LASP1 and CRKL (Figure 4). While phosphorylation is reversible under nilotinib treatment in Ba/F3p210 cells, the TKI-resistant mutant Ba/F3p210^T315I^ cells showed an impaired phosphorylation status (Figure 4, right panel), suggesting that LASP1 is a direct substrate of BCR-ABL.

### LASP1 is not localized within the nucleus in CML cell lines

A predominant nuclear localization of LASP1 is observed in several cancer entities and was reported to correlate with worse long-time survival in breast cancer [18]. To test the subcellular LASP1 expression pattern in CML, Western blot analyses of cytosolic and nuclear fractions from K562, M07e, M07p210, BV173, Ba/F3 and Ba/F3p210 before and after nilotinib treatment were performed.

**Figure 5:**
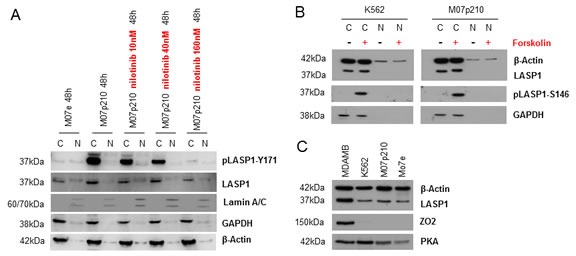
In CML cells LASP1 is not localized in the nucleus (A) Western blot analysis (10% gel) of LASP1 and pLASP1-Y171 in cytosolic (C) and nuclear (N) fractions of M07e and M07p210 leukemia cells before and after nilotinib treatment. Phosphorylation at Tyr-171 by BCR-ABL does not influence cytosolic LASP1 localization. (B) Western blot analysis of LASP1 and pLASP1-S146 in cytosolic (C) and nuclear (N) fractions of M07e and M07p210 leukemia cells before and after forskolin stimulation. Phosphorylation of LASP1 by PKA does not influence cytosolic LASP1 localization. Purity and loading of the fractions were controlled by Western blot for β-actin, the cytosolic marker GAPDH and the nuclear marker Lamin A/C. (C) Western Blot analysis of LASP1, ZO2 and PKA expression in MDA-MB 231 breast cancer cells comapred to K562, M07p210 and M07e leukemia cells. Loading was adjusted to similar ß-actin levels.

**Figure 6:**
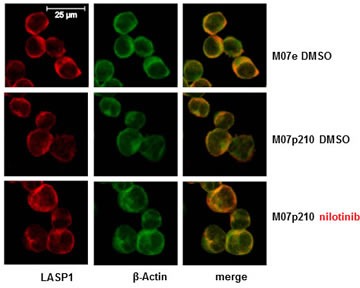
Immunofluorescence of LASP1 in Mo7e and M07p210 cells Cells were treated with or without 160 nM nilotinib for 24 h, fixed, permeabilized and stained for LASP1 (red), actin (green) and merge (yellow). Scale bars 25 μm. No change in cytosolic and membranous LASP1 localization is observed.

Loading of the nuclear fractions was tested by Western blot analysis for Lamin A/C; GAPDH as a solely cytosolic protein served for nuclear purity. As seen from Figure 5A, LASP1 and pLASP1-Y171 are localized exclusively in the cytosol and dephosphorylation of LASP1 by nilotinib treatment has no influence on subcellular LASP1 localization (shown representative for M07e and M07p210 in Figure 5A). These data were validated by indirect immunofluorescence of LASP1 in K562 and M07p210 cells treated with nilotinib or vehicle (shown for M07e and M07p210 in Figure 6).

LASP1 is known to shuttle between the cytosol and the nucleus in a phosphorylation dependent manner [24]. Phosphorylation of LASP1 by protein kinase A (PKA) at serine 146 induces translocation of the protein from the cytoplasm to the nucleus by binding to the zonula occludens protein 2 (ZO2) [24]. We therefore tested i) for the expression of the involved shuttle proteins in CML cells and ii) for a potential nuclear translocation of LASP1 after PKA activation by forskolin. As seen in Figure 5B, phosphorylation of LASP1 at Ser-146 is not inducing any translocation of the protein to the nucleus and besides, the CML cell lines do not express the LASP1 binding shuttle partner ZO2 as do breast cancer cells (Figure 5C).

### pLASP1-Y171 and CRKL are binding partners

It is well established that tyrosine phosphorylated proteins are specifically recognized by SRC Homology 2 (SH2) domains [25]. To determine the binding preferences of different SH2 domains to pLASP1-Y171, we screened a panel of 74 different SH2 domains for preferential binding to a LASP1-Y171 phosphopeptide (Supplementary Figure 2). The corresponding, non-phosphorylated peptide served as control [26]. SH2 domains of the tyrosine kinases ABL1 and 2, BLK, FRK, FYN, LCK, LYN, and YES scored as top binders of which ABL is already known to bind and phosphorylate LASP1 [8, 27]. Among the adaptor proteins carrying SH2 domains, CRKL was identified as the strongest binder suggesting that CRKL is preferentially binding to phosphorylated LASP1 with high affinity.

To verify this predicted interaction, we performed far Western blot experiments by overlaying NC-blotted His_6_-LASP1 and His_6_-pLASP1-Y171 with GST-CRKL and GST-pCRKL. For these experiments, His_6_-LASP1 and GST-CRKL were phosphorylated maximally by active recombinant ABL kinase (see Supplementary Figure 3). Y171 is the only ABL kinase phosphorylation site in LASP1. A second potential tyrosine-kinase consensus sequence at Y186 was excluded earlier by mutational analysis [27].

**Figure 7:**
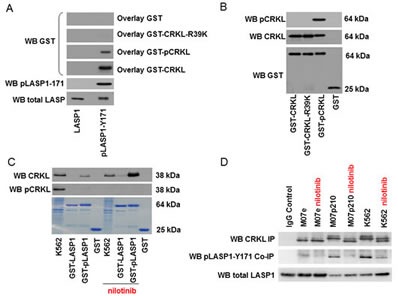
CRKL binds to phosphorylated LASP1 (A) Western blot analysis (10% gel) of bound CRKL to LASP1 and phosphorylated pLASP1-Y171 (pLASP). Blotted LASP1 and pLASP1-Y171 were overlayed with GST (control), GST-CRKL, phosphorylated GST-pCRKL and mutated GST-CRKL-R39K. Binding was detected by GST Western blot. CRKL binds only to phosphorylated pLASP1-Y171. (B) Western blot control of GST-CRKL concentrations used for the overlay experiment in Figure 6A. (C) Western blot analysis (10% gel) of CRKL, pulled-down with GST-LASP1 and GST-pLASP1-Y171 from K562 homogenate. In the lower Coomassie-stained part, used GST-beads and K562 homogenate is shown. Phosphorylation of LASP1 at Tyr-171 results in a faint shift. (D) Western blot analysis (10% gel) of CRKL, immunoprecipitated with anti-CRKL antibody from leukemia cell lines as indicated, and LASP1/pLASP1-Y171, co-immunoprecipitated with CRKL.

As shown in Figure 7A and in accordance to our phosphopeptide binding studies, CRKL and pCRKL, albeit to a much lower extent, bind to phosphorylated LASP1 (pLASP1-Y171). No binding to non-phosphorylated LASP1 is observed. Equal overlay concentrations were confirmed by Western blot (Figure 7B).

To demonstrate that interaction of CRKL with phosphorylated LASP1 is dependent on the SH2 domain of CRKL, the arginine residue in the FLVRES-motif of the SH2 domain of CRKL was mutated to lysine. Mutation of arginine in the FLVRES-motif abolishes the binding of SH2 domains to phosphorylated tyrosine residues [28]. As demonstrated by far Western blot analysis, binding of CRKL-R39K to phosphorylated LASP1 is abrogated (Figure 7A) confirming that CRKL primarily interacts with phosphorylated LASP1 via its SH2 domain.

CRKL-LASP1 binding was further confirmed by pulldown and immunoprecipitation assays. For the pulldown experiments, purified GST-LASP1 and GST-pLASP1-Y171 were incubated with K562 cell lysate partially pre-treated for 48 h with nilotinib to inhibit BCR-ABL kinase and to abolish CRKL phosphorylation. Western blot analysis for CRKL and pCRKL demonstrated only co-pulldown of dephosphorylated CRKL from the nilotinib-treated K562 cells with GST-pLASP1-Y171, while no pCRKL binding is observed in the non-treated cell lysate, neither with LASP1 nor with pLASP1-Y171 (Figure 7C). The faint visible band binding to pLASP1-Y171 in the non-treated K562 cell homogenate is dephospho-CRKL as confirmed by the band-shift at 37 kDa (Figure 7C, upper panel). Similar results were obtained with M07p210 -/+ nilotinib (data not shown). Maximal dephosphorylation of CRKL in nilotinib-treated K562 cells (Figure 7C, middle panel) as well as maximal LASP1 phosphorylation (not shown) was controlled by Western blot and became also apparent in gel shifts in Figure 7C.

Incubation of cell lysate with CRKL specific antibody revealed co-immunoprecipitation of pLASP1-Y171 with CRKL from K562, M07p210 and Ba/F3p210 cell homogenate (all active BCR-ABL) while in cell lysates comprising dephosphorylated LASP1 (Ba/F3 and M07e – no BCR-ABL or K562/nilotinib and M07p210/nilotinib – inactive BCR-ABL) no co-immunoprecipitation of pLASP1-Y171 is detected (shown for K562 and M07p210 in Figure 7D).

In view of the LASP1-CRKL binding results, we also performed immunocytochemistry staining experiments in K562 and M07p210 CML cells to test for LASP1-CRKL co-localization and potential influence on protein level before and after dephosphorylation under nilotinib treatment. As seen in Supplementary Figure 4, independent of their phosphorylation status, both proteins are co-localized at the membrane edges of the cell.

## DISCUSSION

The advent of selective BCR-ABL tyrosine kinase inhibitors revolutionized outcome in CML, and the knowledge about the precise molecular mechanisms of resistance, signal transduction, and disease biology is advancing [29]. Biological markers such as BCR-ABL expression, gene expression profile, CRKL phosphorylation or expression of imatinib transporter proteins have been shown to be useful to predict response to treatment [30]. Notwithstanding, there are still patients who fail in achieving therapeutic goals (i.e. major molecular or complete cytogenetic response) despite continuous TKI therapy. It is thought that leukemia stem cells (LSC) (primitive CML progenitor cells) may not be strictly addicted to BCR-ABL and to be able to bypass BCR-ABL signaling via other pathways when treated with TK inhibitors [29]. The identification and inhibition of these pathways implicated in LSC survival, in combination with conventional therapy, is the new therapeutic ambition in CML treatment to overcome imatinib resistance and eradicate minimal residual disease (MRD) in CML [29].

With LASP1 we identified a novel BCR-ABL substrate in CML. LASP1 is significantly (p < 0.0001) overexpressed in CML cells compared to normal tissues, bone marrow-derived cells, and other hematological malignancies (Figure 1A). Interestingly, during blast crisis, LASP1 mRNA decreases compared to chronic phase as shown in a recent microarray analysis [9]. However, no data for control mRNA is available for this microarray data set [9]. Therefore it remains elusive, whether LASP1 levels revert to normal during blast crisis or stay up-regulated, albeit below chronic phase. Concerning the publicly available CML datasets used in the current study (Figure 1A), clinical annotation of disease stages were either not available or were inconclusive for the majority of samples, which precluded robust statistical analysis of LASP1 expression in subgroups according to CML phases.

LASP1 is phosphorylated at Tyr-171 by BCR-ABL kinase and in turn interacts with the SH2 domain of CRKL, the major BCR-ABL substrate in CML [31]. These data suggest that LASP1 may have an important function for the biology of this disease.

Phosphorylation at Ser-146 and binding to ZO2 is essential for LASP1 to translocate from the cytosol to the nucleus and to transmit extracellular signals [24]. This signaling pathway seems to be modified in CML. In contrast to cells in a solid tissue or tumor, non-adherent circulating CML cells do not express the indispensable LASP1 shuttle partner and tight junction protein ZO2 (Figure 5C), thus explaining the lack of nuclear LASP1 in these cells.

Apart from its involvement in BCR-ABL signaling, LASP1 expression is subsequently induced by sonic Hedgehog (SHH) pathway [32] SHH hyperactivation correlates with CD34 expression, increases from chronic to blast phase [33] and is one out of four major pathways that have been implicated in the survival of LSC representing a potential therapeutic target [29].

An additional fact for the importance of LASP1 in CML is the observed binding of LASP1 to chemokine receptors (CXCR) 1-4. CXCR4 mediates the migration of hematopoietic cells to the bone marrow and plays a key role in the interaction between leukemic and stromal cells. BCR-ABL positive cells show an impaired and downregulated expression of CXCR4 [34]. Binding of LASP1 to CXCR4 requires phosphorylation of LASP1 at Ser-146 PKA [35]. Whether phosphorylation of LASP1 at Tyr-171 has an effect on CXCR binding has not yet been studied.

It is assumed that upon Tyr-207 phosphorylation of CRKL by ABL kinase, an intracellular binding of the SH2 domain occurs, preventing the SH2 domain from interacting with other phosphorylated ligands [36]. Our pulldown and overlay experiments with CRKL and pCRKL, in combination with LASP1 and pLASP1, confirmed these data. Binding of phosphorylated LASP1 predominantly occurs with the non-phosphorylated form of CRKL. Both proteins are adaptor proteins co-localizing at the plasma membrane and both are involved in a number of biological processes such as cell proliferation, cell adhesion, migration, and regulation of gene expression [10, 37]. In breast cancer cells, phosphorylation of LASP1 by ABL kinase occurs during apoptotic processes and is associated with the loss of LASP1 localization to focal contacts [8]. However, LASP1 phosphorylation at Tyr-171 has also been implicated in integrin mediated SRC signalling [27]. Indeed, fibrinogen-activated SRC kinase induced cell spreading, development of lamellipodia and translocation of cytosolic LASP1 to focal contacts [27]. A similar process has been observed for the association of CAS to CRKL [38, 39]. Phosphorylation of CAS by SRC promotes binding to CRKL, followed by translocation of CRKL to focal contacts and activation of small G-proteins [38, 39]. Therefore we propose that under physiological conditions LASP1-CRKL binding is regulated by SRC kinase-mediated phosphorylation of LASP1 while in CML constitutive phosphorylation of CRKL and LASP1 by BCR-ABL abrogates the interaction between these proteins, which may impair cell adhesion and migration. In breast cancer, CRKL and LASP1 were shown to be overexpressed and to correlate with tumor growth and progression [12, 13, 16, 37, 40]. Similar observations were made in glioblastoma. CRKL knockdown in glioblastoma cell lines significantly reduced wound healing and invasion [41].

In our Western blot analyses of patient-derived blood samples before TKI treatment, we observed irregular ratios between β-actin and LASP1 or CRKL levels, respectively (Figure 2). These irregular ratios equalizes after nilotinib therapy – most likely due to a more physiological blood cell population after successful response to TKI treatment. However, β-actin levels do not correspond to white blood cell concentrations. We were not able to reliably detect BCR-ABL and ABL kinase in lysates of mature cell compartments from CML patients. This is very likely based on the fact that BCR-ABL and ABL are rapidly degraded under our neutral Triton X-100 lysis buffer conditions while other proteins are not. The degree of degradation depends on hydrolase activity and can be prevented by an acidic pH-value [42].

The comparison between lysates derived from cell culture, peripheral blood of healthy donors and CML patients needs to be interpreted in consideration of their composition, i. e. different cell constituents. Due to the higher homogeneity of cell lines in culture, cell culture experiments reflect more directly the influence of TKI. This TKI effect is less apparent with an increasing amount of “contaminating” normal cells in patient-derived blood samples (see Table 1, BCR-ABL expression before and after therapy). Accordingly, no LASP1-phosphorylation was detected in patients responding to TKI treatment. Results in this cohort reflect the observations made in non-CML control samples.

In summary, our data suggest a strong functional link between LASP1, CRKL and BCR-ABL tyrosine kinase, therewith explaining and supporting the bioinformatic data by Yeung et al [9], who identified LASP1 as a new marker for CML progression. The precise biological and clinical implications of this interaction are subject of ongoing studies.

## METHODS

### Microarray analyses

Publicly available gene expression data of n = 1322 individual samples were retrieved from the Gene Expression Omnibus (GEO) and the Array Express platform hosted at the EBI (http://www.ebi.ac.uk/arrayexpress/). Accession numbers: GSE3526 n = 353 normal tissues; GSE32719, GSE24739 n = 35 CD34^+^ hematopoietic stem cells (HSCs); GSE2125 n = 45 macrophages; GSE7158 n = 26 monocytes, GSE31048 n = 24 B cells, GSE6338 n = 20 T cells; GSE34733, GSE17855, GSE35784 n = 404 AML; GSE14671, GSE17480, GSE24739, GSE33075, E-MEXP-862, GSE13159 n = 187 CML; GSE28460 n = 98 B-precursor ALL; GSE39671 n = 130 CLL). All data were generated on Affymetrix HG-U133plus2.0 microarrays. Expression data were manually revised for their correct annotations and simultaneously normalized by Robust Multichip Average (RMA [43] using custom brainarray (v18 ENTREZG) CDF files yielding one optimized probe-set for each gene corresponding to the ENTREZ gene ID as described elsewhere [44, 45]. Additionally, gene expression data were adjusted for potential batch effects using ComBat [46, 47]. Statistical significance levels between groups were calculated by Mann-Whitney test using Prism 5 (GraphPad software).

### Cell culture conditions

The murine interleukin 3 (IL-3)-dependent pre-B lymphoid cell line Ba/F3, the transformed growth-factor independent Ba/F3p210 cells expressing BCR-ABL, the human granulocyte-macrophage colony-stimulating factor (GM-CSF)-dependent megakaryoblastic cell line M07e, the human CML cell line BV173, human erythroleukemic cells HEL and the human acute myeloid leukemia cell line MOLM-13, were grown in RPMI 1640 supplemented with 2% L-Glutamine (Gibco BRL, Wiesbaden, Germany), 20% heat-inactivated fetal-bovine serum (FBS, Gibco BRL) without antibiotics. Media for growth of Ba/F3 cells and M07e cells were supplemented with 10 ng/mL IL-3 and GM-CSF (Sigma-Aldrich, Taufkirchen, Germany), respectively.

The CML blast crisis cell line K562, the human acute monocytic leukemic cell lines MV4-11 and THP-1, the human T-cell leukemia cell line Jurkat, the human monocyte-macrophage cell line U-937, the human acute promyelocytic leukemia cell line NB4, the human B-cell lymphoma cell line DOHH-2, the human acute lymphoblastic leukemia CCRF-CEM the transformed growth-factor independent M07p210 cells expressing BCR-ABL and the TKI-resistant Ba/F3-BCR/ABL mutant cell line Ba/F3p210^T315I^ were maintained in RPMI 1640 (Gibco BRL) with 2% L-Glutamine, 10% FBS. The human breast cancer cell lines MCF-7 and BT-20 as well as the human epidermoid cancer cells A-431 and the human cervical cancer cell line HeLa were grown in Dulbecco's Modified Eagle's Medium (DMEM) (Gibco BRL) and 10% FBS. All cell lines were cultivated in a 5% CO_2_ atmosphere at 37°C in a fully humidified incubator. The day before each experiment, medium was changed to fresh culture media containing 20% FBS.

### Provenience and preparation of human blood samples

All procedures were performed with IRB approval and complied with relevant national laws, institutional guidelines, and the declaration of Helsinki. All donors gave written informed consent. Peripheral blood from three healthy donors and five patients newly diagnosed to suffer from CML in chronic phase was drawn into EDTA tubes (Sarstedt, Nuembrecht, Germany). Diagnosis was confirmed by blood and bone marrow smears as well as multiplex PCR for BCR-ABL transcripts [48] (see below). Patients 1-4 were treated with 600 mg nilotinib daily (300 mg every 12h), patient 5 was treated with 45 mg ponatinib daily (for detailed patients’ characteristics see Table 1). Whole blood samples were lysed with five volumes of hypotonic erythrocyte lysis buffer (Qiagen, Hilden, Germany) from at least 20 ml of peripheral blood. After 10 min incubation at room temperature (RT) and dual centrifugation (472 × g), the plasma-free leukocytes were resuspended in PBS at a concentration of 2 × 10^7^ cells/ml. Prepared cells were immediately used for further analysis.

### Inhibition of BCR-ABL

BCR-ABL activity was blocked using nilotinib (Cayman Chemical, Ann Arbor, MI, USA); 10 mM stock solution in dimethyl sulfoxide (DMSO) (Sigma-Aldrich, Taufkirchen, Germany) kept at -20°C.

### Preparation of nuclear and cytosolic cell fractions

For preparation of cell fractions cells were harvested and washed twice in PBS. Isolation of nuclei and cytosol was carried out using NE-PER nuclear and cytoplasmic extraction reagents (Pierce, Bonn, Germany) following the manufacturer's instructions. Purity of the fractions was controlled by probing Western blot membranes for the nuclear marker proteins Lamin A/C (sc-6215, Santa Cruz) and the cytoplasmic marker protein GAPDH (sc-20357, Santa Cruz).

### Western blot analysis

PBS-washed cells were lysed on ice at 1 × 10^7^ cells per 100 μl of 1% Triton X-100-based lysis buffer containing 20 mM HEPES, 150 mM sodium chloride, 10 mM EDTA, 2 mM EGTA, 10 mM tetrasodium pyrophosphate, 50 mM sodium fluoride, (all Sigma-Aldrich). Shortly before lysis 1 mM sodium orthovanadate (freshly boiled) and fresh protease inhibitors (Sigma-Aldrich) were added. Total protein was quantified using the Bradford assay (Biorad, Munich, Germany). Protein lysates were subjected to SDS-PAGE and blotted onto PVDF membrane. Equal amounts of protein were analyzed by immunoblotting.

The antibody against ABL (#554148) was purchased from BD Pharmingen™ (Heidelberg, Germany). LASP1 antibody was described previously [4]. Monoclonal rabbit pLASP1-Y171 specific antibody (1:2000) was generated by EB (Supplementary Figure 1).

Antibody against ZO2 was purchased from New England Biolabs (Frankfurt, Germany). Catalytic *PKA* subunit was detected with a specific antiserum kindly provided by Dr. G. *Schwoch* (1:4000; *University* of *Göttingen*, Germany).

Western blot analysis of p/CRKL was performed as described [7]. CRKL antibody was purchased from Santa Cruz (sc-319, Santa Cruz, Heidelberg, Germany) and detects the phosphorylated 38 kDa protein and the non-phosphorylated 37 kDa protein by separate band shifts in a gradient gel. pCRKL-Y207 (ab52908) and PY20 (ab10321) antibodies were purchased from Abcam. Equal protein loading was confirmed by documenting β-actin content with the goat polyclonal anti-β-actin antibody (sc-1616, Santa Cruz).

Before overnight incubation with primary antibodies, membranes were blocked with 5% dry milk (Biorad, Munich, Germany) in TBS-T for 1h at RT. Immunoblots were probed with a secondary horseradish peroxidase conjugated antibody purchased from Santa Cruz and developed by using the enhanced chemiluminescence reagent (GE Healthcare, Freiburg, Germany). Chemiluminescence images were taken using the Fujifilm LAS-3000 system (Fuji, Düsseldorf, Germany) and quantified by the AIDA Image Analyzer.

### Molecular cloning and expression of CRKL, LASP1 and CRKL-R39K

For PCR amplification of CRKL, gene-specific primers were designed based on the published human cDNA sequence (GenBank Access. No. CAG30309.1):

CGCGTCGACTCATGTCCTCCGCCAGGTTC (forward primer with *Sal*l restriction site) and CGCGCGGCCGCTCACTCGTTTTCATCTGGGT (reverse primer with *Not*I restriction site). Full length CRKL cDNA was cloned into the *Sal*l and *Not*I sites of pGEX4T-1 (GE Healthcare) to generate GST-CRKL fusion gene.

To generate GST-LASP1 fusion gene the following oligonucleotides were used: AATGGATCCATGAACCCCAACTGCG-CCCGGTGCGGCAAG (sense, with *Bam*HI restriction site) and CGGGAATTCTCAGATGGCCTCCACGTA GTTGGCCGGCA (antisense, with *Eco*RI restriction site).

For generating His_6_-tagged LASP1 we used the oligonucleotides CGCCATATGAACCCCAACTGCGCCCGG (forward primer with *Nde*I restriction site) and CGCCTCGAGTCAGATGGCCTCCACGTAGT (reverse primer with *Xho*I restriction site). In order to get the His_6_-tagged LASP1, full length cDNA (GenBank Access. No. X82456) was cloned into the *Nde*I and *Xho*I sites of pET-28b vector (Novagen/Merck Millipore).

To exchange arginine for lysine at position 39 of CRKL protein, amino acid sequence appropriate primers were designed: CACGGTATGTTCCTCGTCAAGGATTCTTC CACCTGCCCT (sense) and AGGGCAGGTGGAAGAATCCTTGACGAGG AACATACCGTG (antisense) (www.stratagene.com).

The recombinant vector pGEX4T-1-CRKL served as template for mutagenesis-PCR. The PCR was performed using QuickChange Site-directed Mutagenesis Kit (Agilent Technologies, Böblingen, Germany) according to supplier instructions.

All constructs were verified by DNA sequence analysis. All primers were synthesized by Eurofins MWG Synthesis GmbH (Ebersberg, Germany).

### RNA extraction, reverse transcription and quantitative real-time polymerase chain reaction

Total leukocyte RNA was extracted from at least 2 × 10^7^ peripheral leukocytes using Trizol reagent (Invitrogen, Karlsruhe, Germany) according to the manufacturer's instructions after lysis of red blood cells. Messenger RNA was reversely transcribed into complementary DNA (cDNA) using random hexamers as described before [49]. The assessment of BCR-ABL transcript level was performed using qRT-PCR as previously described [50]. BCR-ABL transcript levels were expressed as a ratio BCR-ABL/ABL according to the International Scale, IS [51].

### Protein expression and purification

CRKL, CRKL-R39K and LASP1 were expressed as GST-fusion-proteins in BL21DE3cells (NEB, Frankfurt am Main, Germany). After lysis (50 mM Tris, 1 mM EDTA, 100 mM NaCl, 0.1% Triton X-100, pH 8.0 and protease inhibitor cocktail) and sonication, fusion proteins were affinity-purified using gluthatione-sepharose 4B (GE Healthcare, Freiburg, Germany) and eluted with 60 mM glutathione (Roche, Mannheim, Germany).

For His_6_-tagged LASP1, the recombinant pET28b-LASP1 vector was transformed into BL21DE3 *E. coli*. Bacteria were lysed (50 mM Tris, 0.6 mM EDTA, 300 mM NaCl, 0.1% Triton X-100, pH 8.0 and protease inhibitor cocktail), sonicated and His_6_-LASP1 was affinity-purified using HisTrap HP affinity columns (GE Healthcare) following the instructions of the supplier.

Protein levels were up-concentrated using Centricon^®^ Centrifugal Filter Devices YM-10 / YM-30 (Amicon, Merck Millipore). Proteins are stable for at least 6 weeks at 4°C.

### Phosphorylation of CRKL and LASP1

LASP1 and CRKL were phosphorylated by recombinant active ABL kinase (BPS Bioscience, San Diego, CA, USA) according to the suppliers recommendations at 30°C in phosphorylation buffer (5 mM Tris, 1 mM MgCl_2_, 0.1 mM DTT and 100 μM ATP). For maximal phosphorylation (Supplementary Figure 3), LASP1 had to be incubated with ABL kinase for at least 2h and CRKL for at least 4h (overnight). To block kinase activity in the subsequent experiments, 20fold excess of nilotinib (Cayman Chemical, Ann Arbor, MI, USA) related to ABL kinase concentration was added (Supplementary Figure 3).

Phosphorylation was visualized by Western blot analysis with phosphotyrosine antibody PY20 (1:1000), pCRKL (1:5000) or monoclonal rabbit pLASP1-Y171 specific antibody generated in-house (Supplementary Figure 1) followed by secondary horseradish peroxidase-coupled goat-anti-mouse or goat-anti-rabbit IgG antibody (1:5000) (Biorad, Munich, Germany).

### Overlay (Far western blot)

0.2 μg of His_6_-LASP1 and His_6_-LASP1 phosphorylated by ABL kinase were blotted on nitrocellulose by semi-dry technique. To minimize background binding, membranes were incubated with 5% dry milk in TBS-T for 2h at RT. Membranes were overlaid overnight at 4°C with 1 μg GST-tagged proteins. After three washing steps with TBS-T, membranes were incubated with GST-antibody (ImmunoGlobe, Himmelstadt, Germany) 1:1000 in 5% dry milk in TBS-T followed by secondary horseradish peroxidase-coupled goat-anti-rabbit IgG antibody (1:5000) (Biorad) and enhanced chemiluminescence reagent (GE Healthcare).

### Immunoprecipitation

For immunoprecipitation 1 × 10^7^ cells were lysed as described above in 500 μl of lysis buffer. After centrifugation (14000 × g for 15 min) the supernatants were discharged. Immunoprecipitation was performed by incubating of 500 μl supernatant with 2 μg of the p/CRKL antibody for 2 h at 4°C followed by incubation with protein A-Sepharose (50 μl) under continuous rotation over night at 4°C. The immunocomplex was washed three times with ice-cold lysis buffer, once with 0.5 M LiCl and again with lysis buffer and prepared for SDS–PAGE as described above.

### Pulldown experiments

Cells were lysed with 1% Triton X-100-based lysis buffer described above. After centrifugation (14000 × g for 15 min) the supernatants were incubated for 2 h with 50 μl GST control beads or GST-tagged proteins. Beads were washed three times with PBS and analyzed by SDS–PAGE and Western blot as described above

### Phosphopeptide binding assay

LASP1 peptides (biotin-Ahx-GDGDGGGDDPVpYQQPQQ) corresponding to aa position 169-176 of human LASP1 were synthezised by Biosynthan (Berlin, Germany). The peptides carried a biotin and an aminohexane (Ahx) spacer at the N-terminus followed by a GDGDGGGDD spacer to improve binding. 500 fm of peptide/well were immobilized in 0.1% BSA/PBS to streptavidin coated 96-well microtiter plates (Roche, Mannheim, Germany) for 1h at RT. After washing with 0.1% TBS-T, free streptavidin binding sites were quenched with 1 mM biotin for 30 min at RT and plates were blocked overnight at 4°C in 5% blocking solution (Roche) in the presence of 100 μM sodium orthovanadate. After washing (3 times in 0.1% TBS-T), peptides were incubated in a final volume of 200 μl for 1h at RT with 50 ng/well of SH2 domain precomplexed with streptavidin-HRP (Pierce) as previously described [52]. Plates were washed (8 times) with 200 μl 0.1% TBS-T, 0.1% of ABTS (Roche) was added, plates were incubated for 10 to 30 min at 37°C and OD was measured on a microplate reader at 405 nm. All binding reactions were performed in duplicate.

### Immunofluorescence and confocal laser scanning microscopy

For confocal laser scanning microscopy (CLSM) 3,5 × 10^4^ cells were fixed by adding 10% formalin directly into the culture medium, for a final concentration of 5% which is equivalent to 2% paraformaldehyde. After 10 min incubation at 4°C, cells were centrifuged and washed twice with cold PBS at 1000 × g for 2 minutes at 4°C. Samples were then transferred into a centrifugation chamber with releasable fixing plates and centrifuged for 10 min at 140 × g and RT to generate cytospins. Sections were rinsed in 100% methanol for 5 min at -20 C to perforate cell membranes, air dried, and incubated in 5% BSA in 1 × TBS-T (7.4 mM Tris base; 43.5 mM Tris HCl; 150 mM sodium chloride; 0.1% Tween 20) for 5 min at RT. Subsequently, slices were stained with affinity-purified rabbit polyclonal LASP1 antibody (diluted 1 μg/ml) [19], mouse monoclonal CRKL antibody (1:200; Merck Millipore, Darmstadt, Germany) and Oregon green phalloidin antibody (1:50; Invitrogen, Karlsruhe, Germany) followed by secondary Cy3-labeled anti-rabbit antibody (1:500, Dianova, Hamburg, Germany). Fluorescence recordings were performed using a confocal laser scanning microscope (LSM 510; Carl Zeiss, Jena, Germany) or an inverted microscope (Axiovert 135; Carl Zeiss). An Axiovison software tool (Carl Zeiss) was used for quantitative analysis.

### Author contributions

Conception and design: JF, EB and AH. Provision of study material or patients: JF, PL and AH. Bioinformatic analyses: TG. SH2 profiling: PN and HS. Performance of experiments: JF, EB and CK. Molecular patient characterization: JZ and JC. Manuscript writing and approval: all authors.

## SUPPLEMENTARY MATERIAL AND FIGURES


