# Supplementary material for: LASP1 is a novel BCR-ABL substrate and a phosphorylation-dependent binding partner of CRKL in chronic myeloid leukemia
Source: Oncotarget. 2014 Jun 7;5(14):5257–71. doi: 10.18632/oncotarget.2072 (PMC4170624; doi:10.18632/oncotarget.2072)
Supplement: Supplementary file 1 [file oncotarget-05-5257-s001.pdf]

LASP1 is a novel BCR-ABL substrate and a phosphorylation-dependent binding partner of CRKL in chronic myeloid leukemia

Supplementary Material

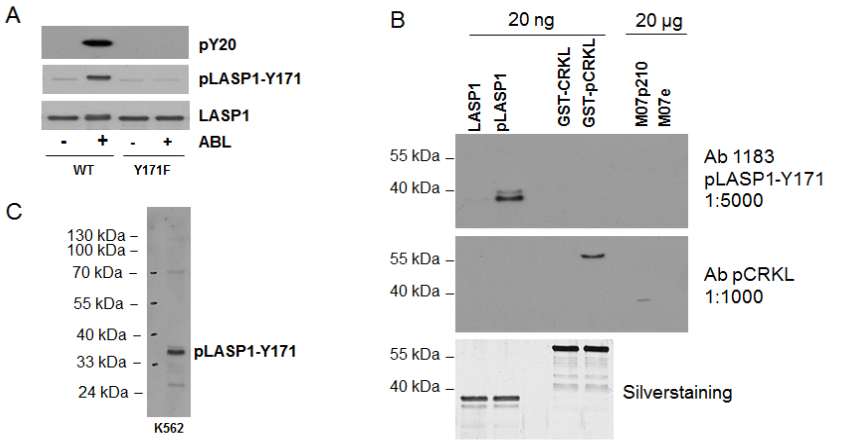

Supplementary Figure 1: Specificity of the antibody recognizing phosphorylated pLASP1-Y171. A: Specificity was tested in Western blots with recombinant wild type GST-LASP-1 and mutant GST-LASP1-Y171F (62 kDa), both phosphorylated with purified recombinant ABL-kinase. The self-generated antibody pLASP1-Y171 does not recognize LASP-1 in its non-phosphorylated state while a strong signal is detected after LASP1 phosphorylation by ABL-kinase. When using the non-phosphorylable mutant LASP1-Y171F, no phosphate incorporation is seen. When reblotting the NC with the unspecific pY-antibody PY20, no additional phosphorylation of pLASP1-Y171 is observed, thus proofing Tyr-171 being the only LASP1 phosphorylation site for ABL-Kinase. B: Western blot cross checking of 20 ng GST-pCRKL and pLASP with both phospho-antibodies (Ab pCRKL and Ab pLASP1-Y171) confirmed specificity of both antibodies. C: Western blot with pLASP1-Y171 antibody in K562 cell homogenate. The antibody recognizes only one band with the correct size of 38 kDa.

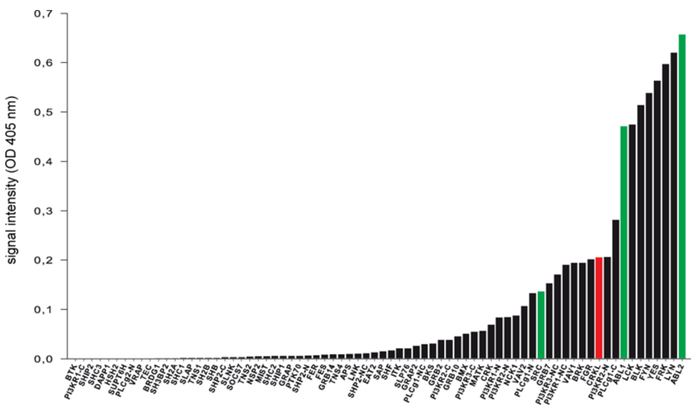

Supplementary Figure 2: Binding preferences of different SH2-domains to the pLASP1-Y171 phosphopeptide (PVpYQQPQQ). The N-terminal biotinylated peptides were immobilized on streptavidin-coated microtiterplates and incubated with 74 different SH2 domains pre-complexed with streptavidin-horseradish peroxidase. After extensive washing, the chromogenic substrate ABTS was added and binding was photometrically determined at 405 nm. Binding assays were performed in duplicate. The average variance of all binding reactions was 5.3%. The non-phosphorylated peptide (PVYQQPQQ) served as a background control. Signal intensities were corrected for non-specific binding to the unphosphorylated peptide, interactions between SH2 domains and pLASP-Y171 were sorted by binding intensity and mean values of duplicate measurements are shown. In red is shown the new identified LASP1 binding partner CRKL. The already known interacting partners SRC and ABL kinase are shown in green.

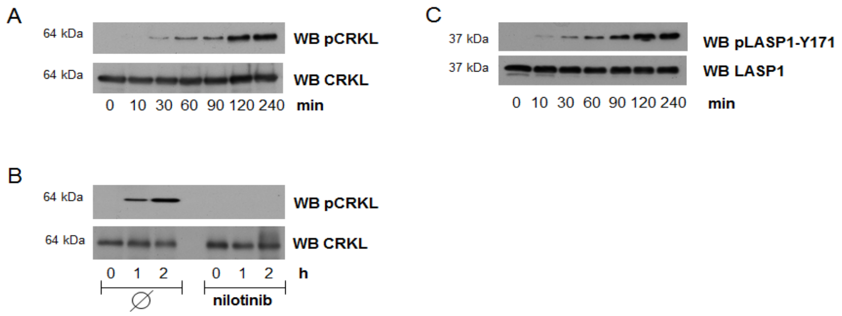

Supplementary Figure 3: Phosphorylation of LASP1 and CRKL by ABL-kinase
A: Time-dependent GST-CRKL phosphorylation (10 µg in 200 µl) by ABL-kinase (0.5 µg).
B: Inhibition of GST-CRKL phosphorylation (10 µg in 200 µl) by ABL kinase (0.5 µg in 200 µl; 0.1 µM) in the presence of nilotinib (2 µM, 20fold excess).
C: Time-dependent His_6-LASP1 phosphorylation (10 µg) by ABL-kinase (0.5 µg).
After the time-points indicated, 20 µl aliquots were taken and analysed by Western blot.

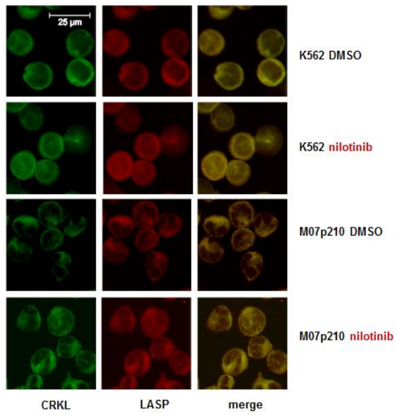

Supplementary Figure 4: ***LASP1 / CRKL immunhistology / co-localization***
Immunofluorescence of LASP1 in M07e, M07p210 and K562 cells. Cells were treated with or without 160 nM nilotinib for 24 h, fixed, permeabilized and stained for LASP1 (red) and CRKL (green). Merge is visualized in yellow. Scale bars 25 µm. LASP1 and CRKL are co-localized at the membrane edges of the cell.
